# Two cases of SMA syndrome after neurosurgical injury to the frontal aslant tract

**DOI:** 10.1007/s00701-022-05466-6

**Published:** 2023-01-10

**Authors:** Kevin Agyemang, Anna Rose, Mustafa El Sheikh, Mutiu Asha, Emanuela Molinari, Natasha E. Fullerton, David Brennan, Athanasios Grivas

**Affiliations:** 1 Institute of Neurological Sciences, Queen Elizabeth University Hospital (QEUH), 1345 Govan Road, Glasgow, G51 4TF UK; 2 School of Medicine, University of Glasgow, University Avenue, G12 8QQ Glasgow, U.K.

**Keywords:** Aphasia, Frontal lobe, Hemiplegia, Neural networks, SMA supplementary motor area, FAT frontal aslant tract

## Abstract

Supplementary motor area (SMA) syndrome is characterised by transient disturbance in volitional movement and speech production which classically occurs after injury to the medial premotor area. We present two cases of SMA syndrome following isolated surgical injury to the frontal aslant tract (FAT) with the SMA intact. The first case occurred after resection of a left frontal operculum tumour. The second case occurred after a transcortical approach to a ventricular neurocytoma. The clinical picture and fMRI activation patterns during recovery were typical for SMA syndrome and support the theory that the FAT is a critical bundle in the SMA complex function.

**Supplementary Information:**

The online version contains supplementary material available at 10.1007/s00701-022-05466-6.

## Introduction

Supplementary motor area (SMA) syndrome, first described by Laplane et al. [10] is typically understood as purely affecting motor function. It is characterised by a paucity of volitional movement and speech, developing minutes or hours after injury to the SMA, with near complete resolutions after a few weeks [10]. However, recent studies have also described pronounced deficits in cognitive function associated with the syndrome [18].

The SMA proper is located posteromedially in the superior frontal gyrus (SFG), immediately anterior to the primary motor area of the lower limb, is bounded inferiorly by the cingulate sulcus [13] but lacks widely accepted anterior and lateral demarcations. The frontal aslant tract (FAT) is a white matter bundle which connects the SFG to the ipsilateral inferior frontal gyrus (IFG) and anterior insular [5, 8, 14, 15].

Meta-analysis of electrophysiology studies suggests a functional–anatomical role for the SMA/pre-SMA complex as a gateway for the executive control network [7, 17]. The FAT connects critical medial parcellations to the lateral area (pre-motor area, IFG, anterior insular and basal ganglia) of the SMA complex across both hemispheres [8]. It has been suggested that SMA syndrome may best be explained as an impairment of this complex [18]. Therefore, we postulate that damage to the FAT may result in deficits beyond language production as previously reported.

We present two consecutive patients who developed SMA syndrome, following surgical insults to the FAT but not SMA proper, to support this view and provide additional insight into the SMA complex.

## Methods

### Setting and time frame

Two consecutive patients who sustained SMA syndrome following surgery at, or through, the lateral prefrontal area but not the SMA proper were identified from six cases of SMA syndrome at a single centre. Patients were identified by the senior author AG, between January 2019 and December 2021, from the records of 60 frontal tumours discussed at neuro-oncology multidisciplinary team meetings. The department provides regional neurosurgical care for 3 million people in the west of Scotland.

### Participants

Adults were included if functional MRI (fMRI), diffusion tractography and pre- and post-operative structural MRI were available. Three cases involving the SMA proper and one with significant pre-operative motor and language deficits were excluded.

Anatomical and functional definitions can be found in the supplementary information (SI) 1.

### Follow-up

Independently ambulant and fluent patients fit to attend their first outpatient review (3-months post-operatively) were classified as recovered. Subsequent 3-monthly reviews followed in the first year unless directed by oncological concerns.

## Results

### Case one

#### [Pre-op]

A right-handed lady in her fifties, with well-controlled epilepsy, presented with new vacant episodes, altered awareness and confusion. Examination was unremarkable with no focal neurological deficits.

**Fig. 1:**
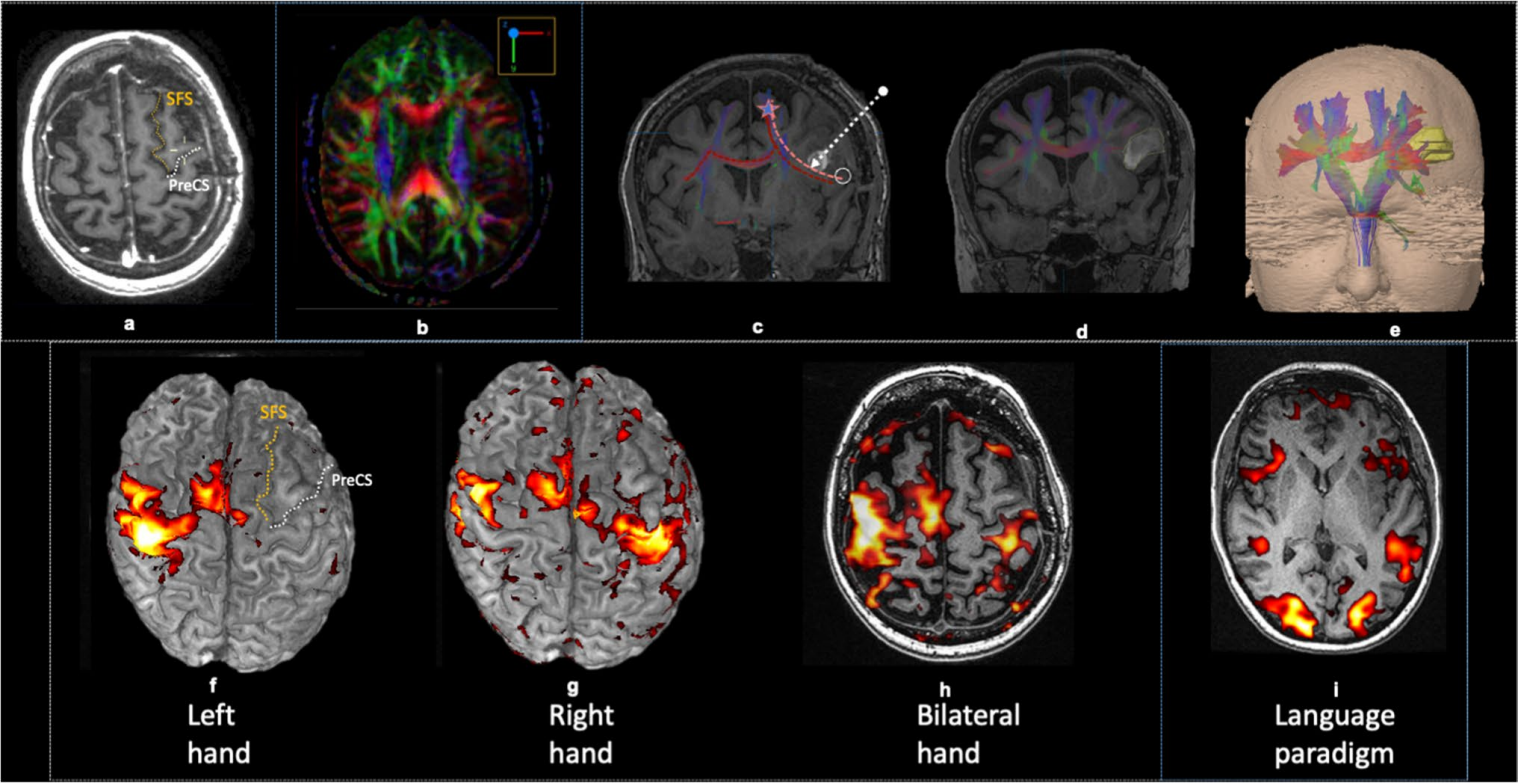
Post-operative imaging: (a) Axial T1-weighted pre-contrast (**a**) showing the resection cavity). (b) Diffusion tensor imaging fractional anisotropy map (**b**) showing altered fractional anisotropy at the level of the lesion, affecting the left aslant tract and arcuate fasciculus. (c) MR-DTI overlaid over coronal volumetric T1-weighted pre-contrast images (**c**) showing relationship of the right FAT (pink dotted line), crossed FAT (red dotted line), and frontostriatal tract (FST) (blue) and the resection cavity (white arrow. (**d**) Sagittal T1-weighted post-gadolinium images of the CST (blue). (e) 3D FAT, cross FAT and CST reconstruction (**e**). SFS superior frontal sulcus, PreCS precentral sulcus, star = SMA, white circle = IFG, Fr.Oper = frontal operculum, Syl.Fiss = sylvian fissure. Blood-oxygen-level-dependent (BOLD) fMRI overlaid on 3D reconstruction (**f**,**g**) and axial (h,i) T1-weighted MR images: (**f**) Right precentral gyrus and SMA haemodynamic activation evoked on left hand motor paradigm. No left SMA activation above threshold. Incidentally, left cerebellar diaschisis observed (not shown here). (**g**) Bilateral precentral gyrus and SMA haemodynamic response evoked during right hand motor paradigm. (**h**) Bilateral precentral gyrus, right but no left SMA response evoked during simultaneous voluntary movement of both hands. (**i**) Bilateral haemodynamic responses in the anatomical Broca, Wernicke’s, and visual association areas evoked during noun–verb language paradigms expected to activate receptive and expressive language areas

MRI revealed extensive high fluid-attenuated inversion recovery (FLAIR) signal changes with heterogeneity in the left IFG, subcentral gyrus and anterior insula (supplementary information SI 2). Post-gadolinium T1-weighted enhancement was observed in the frontal opercular portion, together with calcification on CT and increased uptake on Thallium single-photon emission computerized tomography (SPECT). This raised suspicion of an oligodendroglioma with malignant transformation. Haemodynamic BOLD (Blood-oxygen-level-dependent) fMRI activation in the anatomically expected areas of the left hemisphere and, to a lesser extent, the right suggested language co-dominance with left hemispheric preponderance (Fig. 1i).

Maximal safe resection of the enhancing portion of the tumour was performed via an awake craniotomy with intra-operative neurophysiological mapping of the language areas and motor cortex. The resection was limited by language disturbance (phonemic paraphasia and impaired articulation). This was interpreted as due to disruption of the FAT that normalized when the stimulation was stopped. Clinical and electrophysiological assessment confirmed the integrity of the corticospinal tract (CST) throughout surgery. The patient developed a right hemiparesis at the time of skin closure which progressed to right hemiplegia with associated aphasia immediately after surgery. The injury to the FAT in this case was not intended nor apparent during surgery.

#### [Post-op]

Integrated molecular histopathology revealed the lesion to be a Glioblastoma WHO grade IV, IDH wild type, MGMT methylated. Volitional movements were initially more severely impacted, with occasional reactive motor actions suggesting an intact CST. Language expression improved at day five, with the patient independently ambulant one-month post-operation. The post-operative MRI (Fig. 1a–d) revealed no surgical or ischaemic insults to the SMA region or primary motor area. Reconstructed DTI showed intact CSTs and cross FAT (Fig. 1d), but the ipsilateral FAT, projected to be anteromedial to the surgical cavity, was partially disrupted (Fig. 1b).

Written-word-to-picture-matching (WPM) and picture naming tests improved significantly with only one semantic error after two weeks. Repeat fMRI showed bilateral motor cortex and bilateral SMA hemodynamic activation with right hand movements (Fig. 1f); but only right motor cortex and right SMA activation on left hand movement (Fig. 1e). Interestingly, bilateral hand movements, expected to activate both SMA, only activated the right SMA (Fig. 1h). Bilateral SMA activation on language tasks was unchanged. These findings were interpreted as in keeping with remodelling during recovery to overcome deficits in the SMA complex.

### Case two

#### [Pre-op]

A right-handed woman in her thirties presented with a two-week history of reduced visual acuity and diplopia. Examination demonstrated a left abducens palsy and papilloedema. MRI revealed a ventricular tumour attached to the septum pellucidum, fornix and thalamus causing unilateral ventriculomegaly secondary to obstruction of the left foramen of Monro (supplementary information SI 3). The patient underwent a left frontal craniotomy, with a transcortical transventricular approach to the lesion through the superior frontal sulcus. A near total resection of the tumour was achieved. The patient awoke with aphasia and right hemiparesis affecting only goal-directed actions, as seen in the SMA syndrome.

#### [Post-op]

**Fig. 2:**
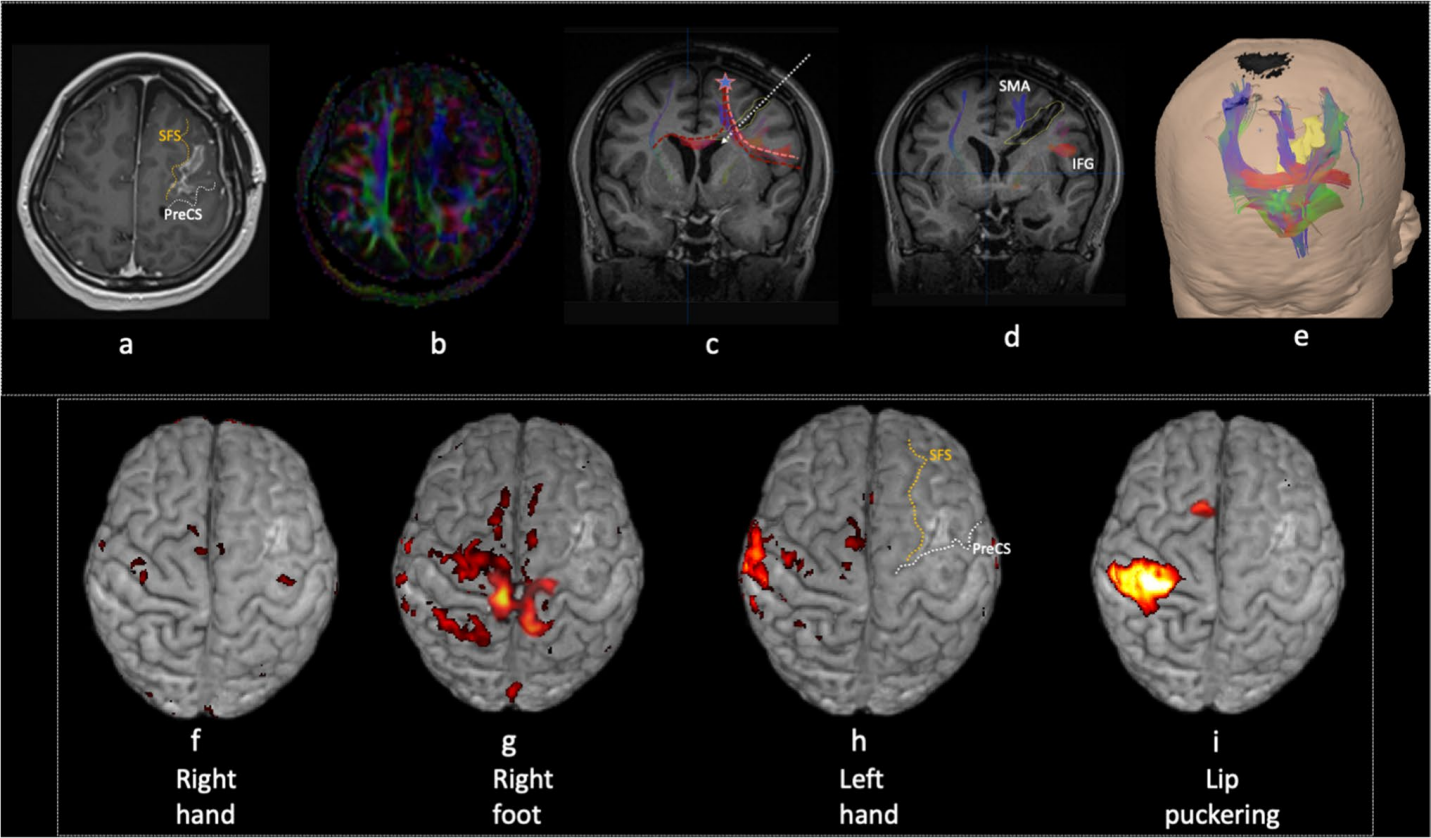
Postoperative imaging: (a) Axial 3D T1-weighted post-gadolinium images (**a**), (c,d) MR-diffusion tensor imaging overlaid over coronal post-contrast 3D T1-weighted images (**c**, **d**) showing relationship of the FAT (pink dotted line), crossed FAT (red dotted line), and frontostriatal tract (FST) (blue) with the resection tract (white arrow). (**b**) Axial fractional anisotropy (FA) map at the level of the resection tract and cavity in the left frontal lobe showing alteration and loss of normal anisotropy. (**e**) 3D relationship between the tumour and frontal aslant tract tractography (red). BOLD fMRI overlaid on 3D reconstruction T1-weighted MR images showing: (**f**) Bilateral precentral regions responses evoked during voluntary right hand motor paradigm. (**g**) Bilateral paracentral lobule and SMA response evoked during voluntary right foot motor paradigm. (**h**) Right precentral gyrus and SMA response evoked during voluntary left-hand motor paradigm. (**i**) Right (unilateral) precentral gyrus and SMA response evoked during voluntary lip puckering motor paradigm. SFS superior frontal sulcus, PreCS precentral sulcus, IFG inferior frontal gyrus

Integrated molecular histopathology confirmed the lesion to be a central neurocytoma (Ki67 3%). Post-operative MRI (Fig. 2a–d) confirmed the surgical tract to the left lateral ventricle traversing the FAT but not the CST or cross-FAT. The SMA, precentral gyrus and IFG were structurally intact.

Two weeks later, she was ambulant with assistance and attempting to mouth words. One-month post-operatively, she was independent ambulant and spoke albeit with impaired fluency. fMRI performed at this stage showed bilateral motor cortex and SMA haemodynamic activation with right hand (Fig. 2f) and foot motor paradigms (Fig. 2g). Activation of  the right precentral gyrus and SMA was seen on left hand motor paradigm (Fig. 2h). Interestingly, lip puckering motor paradigm resulted in unilateral (right) motor cortex and SMA activation (Fig. 2i). The findings were interpreted as compensation of impaired ipsilateral SMA activity by the rest of the complex.

The patient was back to running and able to hold a telephone consultation two-months after surgery. Procedural assessment, WPM, and picture naming were intact, although a mild “staccato’’ quality remained.

## Discussion

We report two consecutive cases of SMA syndrome after disruption to the FAT but preservation of the SMA proper. The clinical picture and fMRI activation patterns during recovery were typical for classical SMA syndrome.

### Functional similarities with SMA syndrome

Parcellations of the SMA have been proposed to affect motor response thresholds through direct connection with the M1 [6]; the disruptions of which may contribute to SMA syndrome. Reports of motor disturbance from surgery around the FAT have been discussed in terms of direct injury to the SMA proper or CST [1, 19].

However, MRI in both cases showed no injury to the SMA proper or CST. Therefore, similarities between the SMA syndrome and injury to the FAT described in the present report (affecting non-stimulus-driven/volition driven behaviour) suggest the syndrome may result from disruption of networks proximal to the SMA-CST connection.

Electrophysiological and tractography studies have shown the FAT to be the main connection between critical dorsomedial parcellations, dorsolateral clusters (including area 55b), operculo-insular and inferolateral parcellations of the SMA complex across both hemispheres [15, 16]. These parcellations are thought to play a central role in language, sensorimotor and cognitive function including in the salience network [3]. Work by Sjoberg et al. [18] supports the theory that SMA syndrome results from impairment of this complex.

The clinical manifestations of injury to the FAT presented here strengthen the theory that it is integral to SMA complex function [3, 4, 16]. Recruitment of the healthy SMA complex during recovery along with impaired activation of the SMA complex during bilateral or alternating tasks, in the present cases, provides further support for this theory [2, 9]. We postulate that FAT injury reduces the efficiency of the large-scale brain networks with parcellations in the SMA complex and results in a phenotype indistinguishable to the classical SMA syndrome.

### Implications for surgery in the pre-frontal region

Preservation of the FAT bundle immediately adjacent to the SMA proper has been shown to reduce the incidence of SMA syndrome in surgical approaches to the posteromedial SFG [3]. Despite most transcortical approaches to the frontal horn traversing the region of the FAT [11], SMA syndrome from FAT injury nevertheless appears to be uncommon. Extensive injury to the FAT as seen in the present series may increase the risk of SMA syndrome. Fortunately, the recovery in our cases supports prior reports that found no significant association between the extent of FAT damage and recovery [3, 11, 12].

## Limitations

The small number of cases, lack of EMG during fMRI and retrospective determination of the FAT reduces the strength of interpretations drawn from these cases.

## Conclusion

The importance of connections between the SMA and lateral pre-motor cortex in SMA syndrome remains poorly understood. We present two cases of SMA syndrome from isolated FAT injury that further our understanding of the SMA complex and provide insights to the optimal surgical corridor for frontal subcortical pathology.

## Supplementary Information

Below is the link to the electronic supplementary material.

Supplementary file1 (DOCX 14 KB)

Supplementary file2 (PNG 6947 kb)

High resolution image (TIFF 6802 kb)

Supplementary file3 (PNG 3685 kb)

High resolution image (TIFF 6802 kb)

## Data Availability

The datasets generated during and/or analysed during the current study are available from the corresponding author on reasonable request.

## Abbreviations

BOLD: Blood-oxygen-level-dependent
CST: Corticospinal tract
CT: Computed tomography
DTI: Diffusion tensor imaging
EMG: Electromyography
FA: Fractional anisotropy
FAT: Frontal aslant tract
FLAIR: Fluid-attenuated inversion recovery
FMRIB: Functional magnetic resonance imaging of the brain
fMRI: Functional magnetic resonance imaging
FSL: FMRIB study library
FST: Frontostriatal tract
IFG: Inferior frontal gyrus
IDH: Isocitrate dehydrogenase
MEP: Motor evoked potentials
MGMT: Methylguanine methyltransferase
SMA: Supplementary motor area
SPECT: Single-photon emission computerized tomography
SI: Supplementary information
WHO: World Health Organization
WPM: Word-to-picture-matching
