# Supplementary material for: Two cases of SMA syndrome after neurosurgical injury to the frontal aslant tract
Source: Acta Neurochir (Wien). 2023 Jan 10;165(9):2473–8. doi: 10.1007/s00701-022-05466-6 (PMC10477090; doi:10.1007/s00701-022-05466-6)
Supplement: Supplementary file 1 — Supplementary file1 (DOCX 14 KB) [file 701_2022_5466_MOESM1_ESM.docx]

# Supplementary information 1

## **Anatomical definitions**

The SMA proper was defined anatomically on volumetric T1-weighted MRI sequences, as extending from the brain vertex to the cingulate sulcus in the superior–inferior direction, and from the precentral sulcus caudally to the vertical anterior commissure line rostrally.

The FAT was reconstructed using two white matter regions of interest (ROIs) and co-registered onto volumetric MR images. The first ROI was traced in the white matter below the ipsilateral SMA/Pre-SMA-complex guided by the anatomical definition described above. A second ROI was traced in the white matter to the inferior frontal gyrus (pars opercularis and pars triangularis) and anterior insula.

**Functional definitions**

Traditional Brodmann eloquent regions, including the SMA, were established by haemodynamic activation on fMRI during motor and language paradigms. Haemodynamic activation resulting in raised blood-oxygen-level-dependent (BOLD) signal was co-registered with the 3D T1-weighted MRI sequences (guided by accepted anatomical definitions including the forementioned for SMA). The paradigms were of a 'box-car” design with alternating active and baseline tasks of 30 seconds duration for words and noun-verb and 24 seconds for stories language paradigms. Finger and foot tapping and lip puckering were used for motor paradigms. The BOLD signal was analysed in a linear model with t-test statistic set to 0.05 on the FMRIB software library (FSL). Further cluster correction was defined by the z-statistic, with z-scores above 3.5 deemed significant.

            Intra-operative electrostimulation mapping of the FAT was performed under Dexmedetomidine awake anaesthesia in case one. A bipolar stimulator delivering a 1ms (60Hz) pulse frequency was used for cortical mapping to determine the superficial boundaries of the resection. A concentric bipolar aspirator was used for subcortical stimulation to identify the deep functional limits of resection. Motor evoked potentials, direct cortical and subcortical were also performed to map and monitor motor and language for case one.

Intraoperative language mapping was assessed with verb and sentence generation tasks. The FAT was delineated as subcortical sites where stimulation consistently and repetitively induced naming error or speech arrest, with normalization of language on cessation.
