# Supplementary figures and images for: Two cases of SMA syndrome after neurosurgical injury to the frontal aslant tract
Source: Acta Neurochir (Wien). 2023 Jan 10;165(9):2473–8. doi: 10.1007/s00701-022-05466-6 (PMC10477090; doi:10.1007/s00701-022-05466-6)

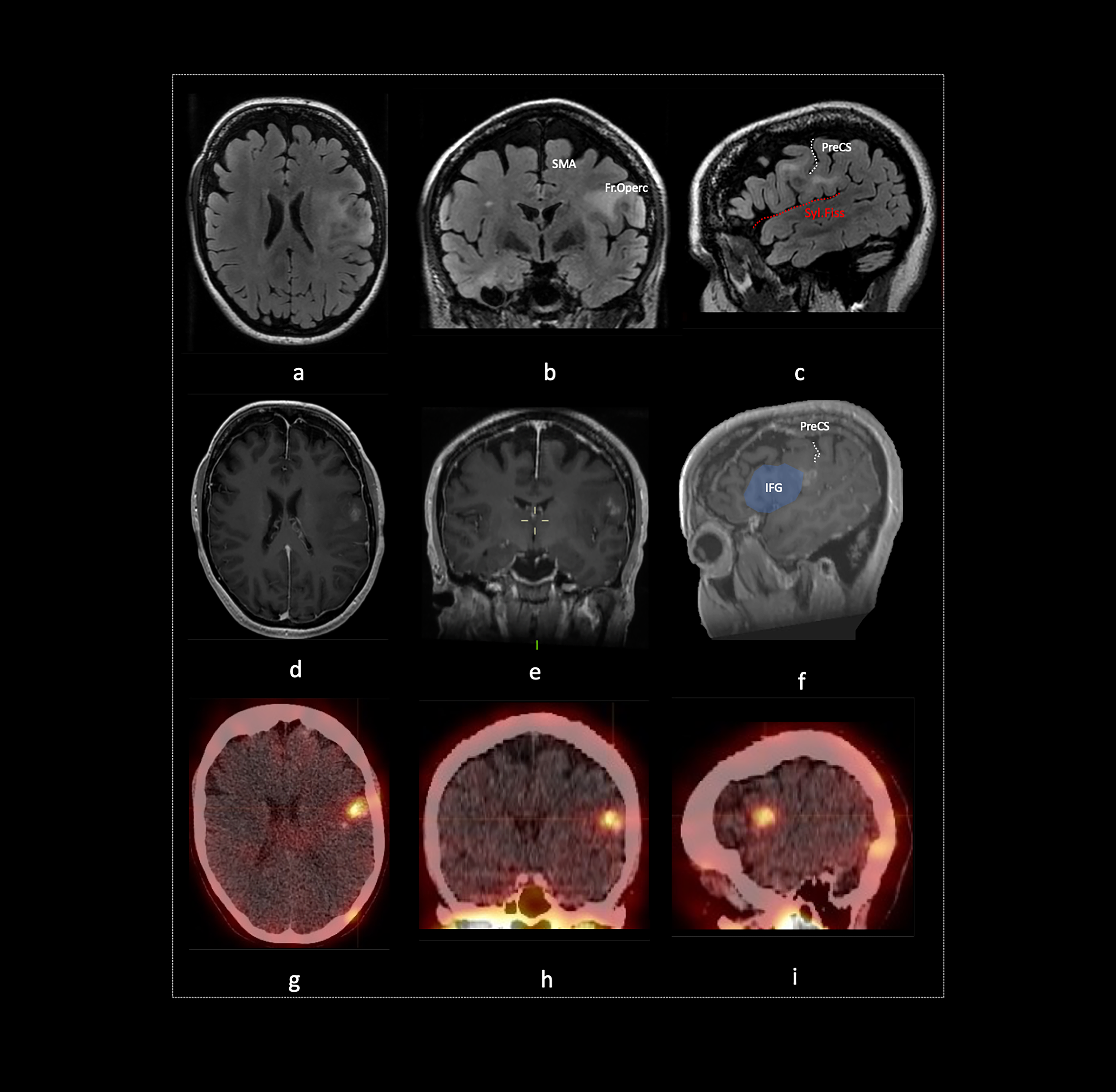

Supplement: Supplementary file 2 — (PNG 6947 kb) [file 701_2022_5466_Fig3_ESM.png]

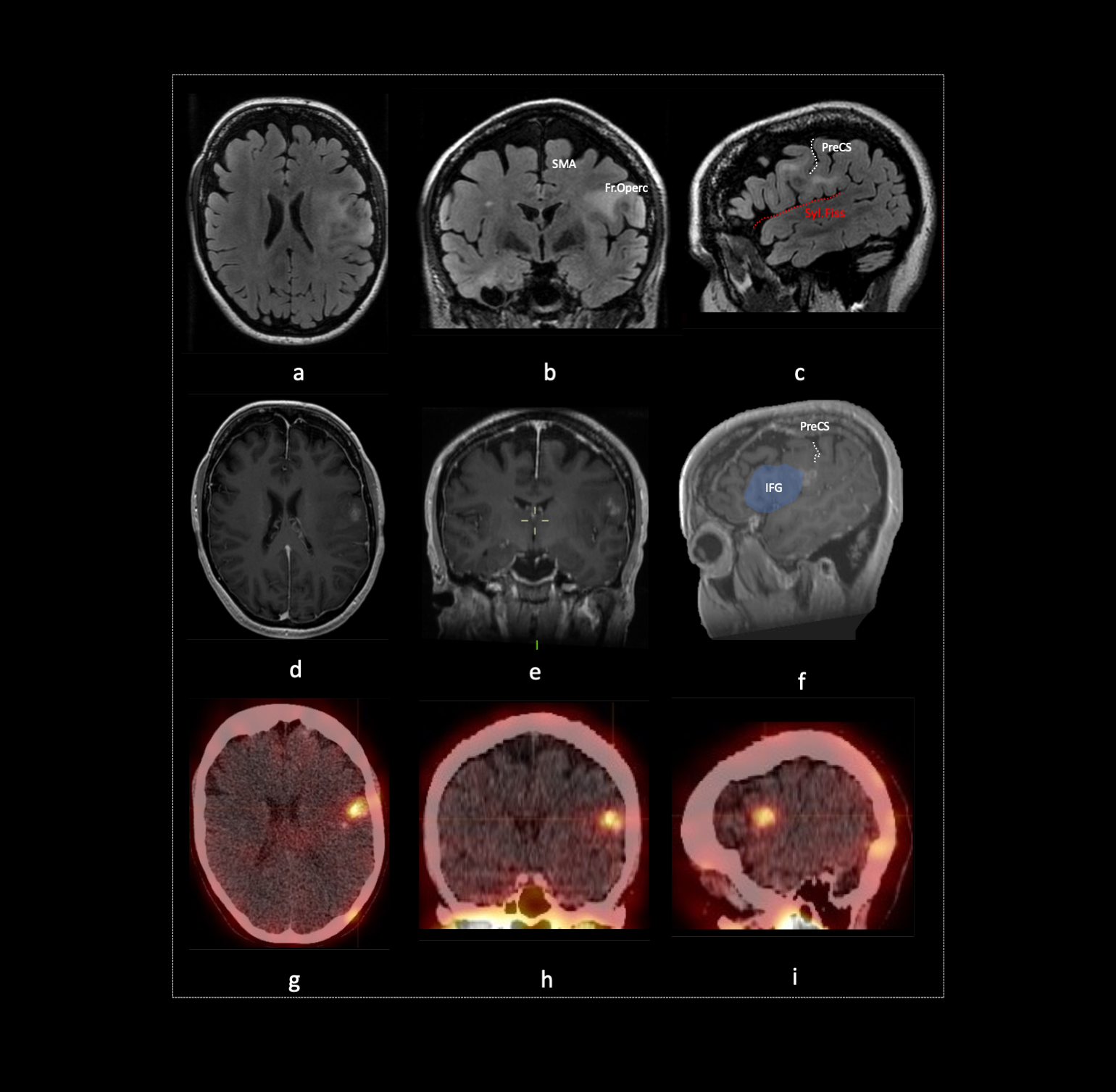

Supplement: Supplementary file 3 — High resolution image (TIFF 6802 kb) [file 701_2022_5466_MOESM2_ESM.tiff]

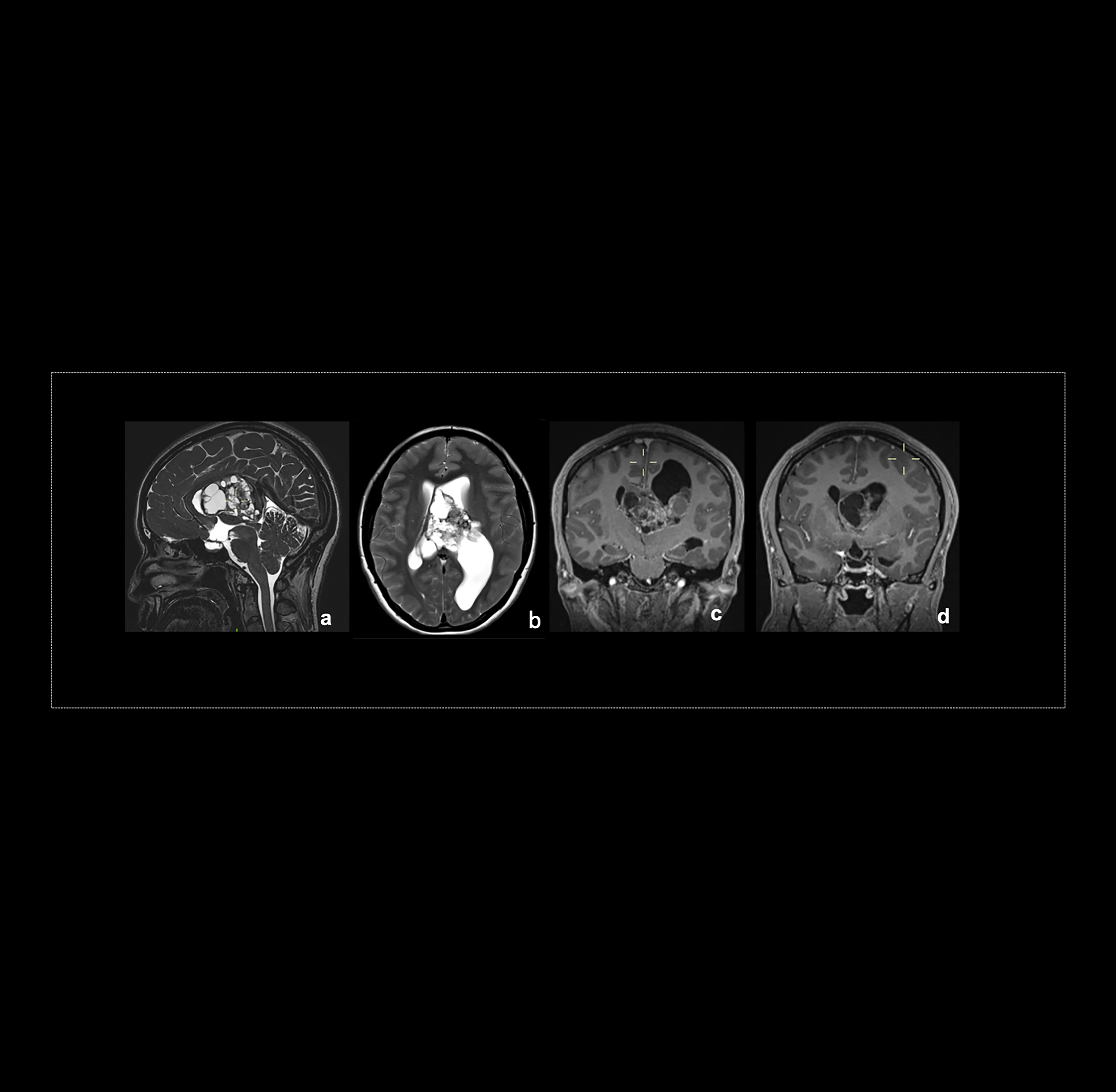

Supplement: Supplementary file 4 — (PNG 3685 kb) [file 701_2022_5466_Fig4_ESM.png]

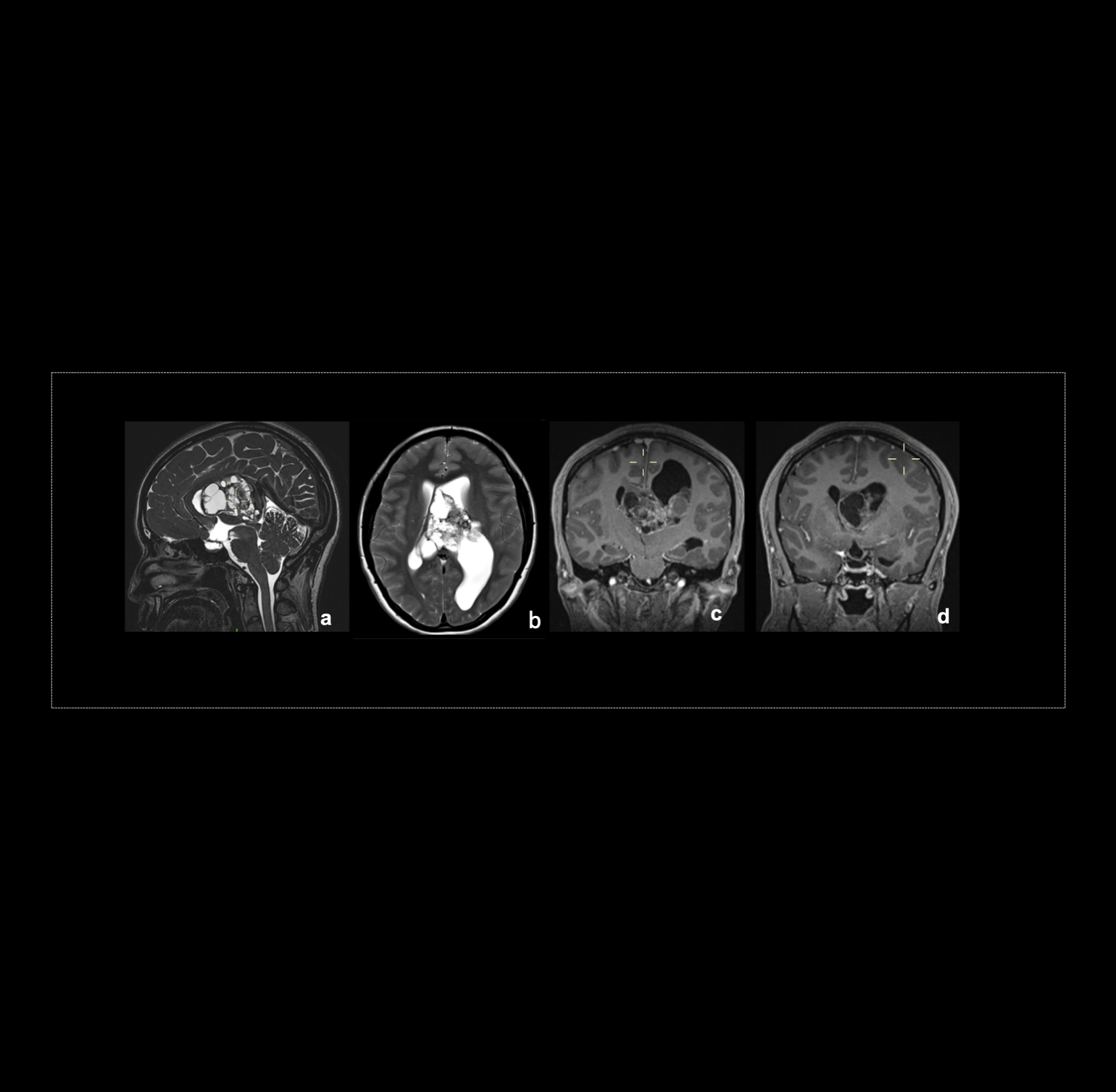

Supplement: Supplementary file 5 — High resolution image (TIFF 6802 kb) [file 701_2022_5466_MOESM3_ESM.tiff]
